# Self-Assembly of Particles on a Curved Mesh

**DOI:** 10.3390/e27010046

**Published:** 2025-01-09

**Authors:** Gabriele Costa, Santi Prestipino

**Affiliations:** Dipartimento di Scienze Matematiche e Informatiche, Scienze Fisiche e Scienze della Terra, Università degli Studi di Messina, Viale F. Stagno d’Alcontres 31, 98166 Messina, Italy; gabriele.costa@studenti.unime.it

**Keywords:** lattice-gas models, spherical boundary conditions, self-assembly

## Abstract

Discrete statistical systems offer a significant advantage over systems defined in the continuum, since they allow for an easier enumeration of microstates. We introduce a lattice-gas model on the vertices of a polyhedron called a pentakis icosidodecahedron and draw its exact phase diagram by the Wang–Landau method. Using different values for the couplings between first-, second-, and third-neighbor particles, we explore various interaction patterns for the model, ranging from softly repulsive to Lennard-Jones-like and SALR. We highlight the existence of sharp transitions between distinct low-temperature “phases”, featuring, among others, regular polyhedral, cluster-crystal-like, and worm-like structures. When attempting to reproduce the equation of state of the model by Monte Carlo simulation, we find hysteretic behavior near zero temperature, implying a bottleneck issue for Metropolis dynamics near phase-crossover points.

## 1. Introduction

A key goal of statistical mechanics is to map out the phase diagram of a many-body system, including attempting to rationalize its behavior in terms of the interaction law. This task usually relies on physical intuition and involves the identification of a few collective variables (so-called order parameters), capturing the nature of each phase at play. However, finding out the order parameters relevant for a specific problem can be difficult, especially when the Hamiltonian contains many couplings and sampling the equilibrium distribution by local Monte Carlo moves proves inefficient (for example, near a phase-transition point, where the simulation can remain trapped in a local free-energy minimum). In this respect, exactly solvable models in one or two dimensions offer a valuable opportunity, since in addition to feeding our insight and knowledge, they provide an illustration of the challenges involved in the study of a statistical system.

To observe non-trivial emergent behavior, the system size taken cannot be small. Clearly, the size should not be too large either, since otherwise no exact computation of the partition function is possible. An easy way to generate unusual spatial organization in a relatively small system is to choose an ambient space with intrinsic curvature, since then the particle interaction will typically be frustrated. To avoid having to be concernedwith boundary effects, a natural choice is a (two-dimensional) system of particles confined on a surface with the topology of a sphere. A further simplification is obtained by discretizing the space, e.g., by taking particles to lie at the nodes/sites of a dense, quasi-regular triangular mesh on the sphere [1]. The combination of a lower dimensionality and discrete particle positions can make the model amenable to an exact treatment, either analytic or numerical.

In this paper, we reconstruct the *exact* equilibrium behavior of a lattice-gas model on a mesh obtained by slightly deforming the vertices of a pentakis icosidodecahedron [2]. For this model a rich interplay can be expected between “phases” with different symmetries. Clearly, in a finite network, genuine phases can only exist at zero temperature (T=0), since at T>0 any phase transition will be smeared out, i.e., replaced by a smooth crossover region (see, e.g., [3]). Self-organization on the vertices of a convex polyhedron has distinctive features not present in a lattice system in flat space. In particular, in a spatial grid having the topology of a sphere, we observe an excess of fivefold vertices over sevenfold ones. As we demonstrate in the following, this does not preclude the onset of low-temperature structures with non-trivial symmetries. For our study we employ the Wang–Landau (WL) algorithm [4,5], allowing us to compute—with negligible error—the density of states of the model, and thus obtain all thermodynamic properties at once.

The plan of the paper is as follows. In Section 2, we introduce the model and explain how its thermodynamic behavior can be worked out exactly. The results are presented in detail and commented on in Section 3. Finally, Section 4 is devoted to conclusions and future perspectives.

## 2. Model and Method

The model under study is a lattice-gas model, meaning that the generic site *i* of the mesh is either occupied (by a “particle”, $c_{i}=1$) or empty ($c_{i}=0$), with i=1,…,M; particles in neighboring sites interact according to a certain Hamiltonian H[c], where $c=\{c_{1},\ldots ,c_{M}\}$ is the set of occupation numbers (microstate). In the present study, the mesh is derived (by a procedure described below) from the vertices of a convex polyhedron called the *pentakis icosidodecahedron* (PID).

**Figure 1:**
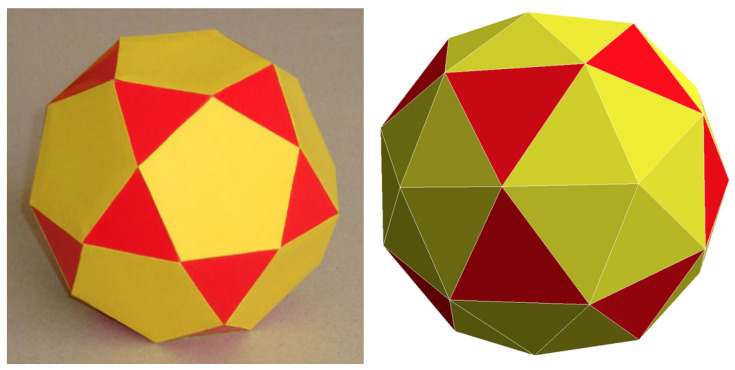
Construction of a PID: (**Left**) The starting point is an ID, a polyhedron with 30 vertices and 32 faces (here is a paper model). (**Right**) The PID is obtained by raising a shallow pyramid on each pentagonal face of the ID (Conway’s kis operation). The resulting number of vertices is 42, while the number of faces is 80 (20 equilateral triangles—the red ones in the picture—and 60 isosceles triangles).

The icosidodecahedron (ID) is an Archimedean polyhedron with faces that are regular triangles and pentagons, meeting in the same pattern around each vertex (see Figure 1, left). It turns out that the vertices of the ID are the midpoints of the edges of a regular icosahedron (or dodecahedron). The PID is obtained by raising a pyramid on each pentagonal face of the ID, thus adding a further 12 vertices (Figure 1, right), thus arriving at a total of 42. The height of the pyramid is chosen such that all PID edges are tangent to the unit sphere (thereby resulting in an almost spherical polyhedron). In the spherical PID, which is the variant considered in the present paper (referred to as PID from now on), the original vertices are projected on the unit sphere, with the result that the polyhedron can now be inscribed in a sphere. The new vertices define the sites of the mesh. However, we emphasize that this projection has no effect on the energy value, in so far as the interaction is only sensitive to the coordination relationships between the sites (the case would be different if the strength of interaction were dependent on the Euclidean distance between the sites).

In the second and third columns of Table 1, we have listed the chord distances separating a reference site (either sixfold or fivefold) from the sites which are closest to it on the PID mesh, using the radius of the circumscribed sphere as the unit of length. If we take the coordination numbers in the triangular lattice for comparison, then we are naturally led to consider the six nearest sites to a sixfold site as all belonging to the first-neighbor shell, despite the distance from the central site not being the same for all. Similarly, we include in the second neighbors of a sixfold site two groups of sites at slightly different distances from it.

**Figure 2:**
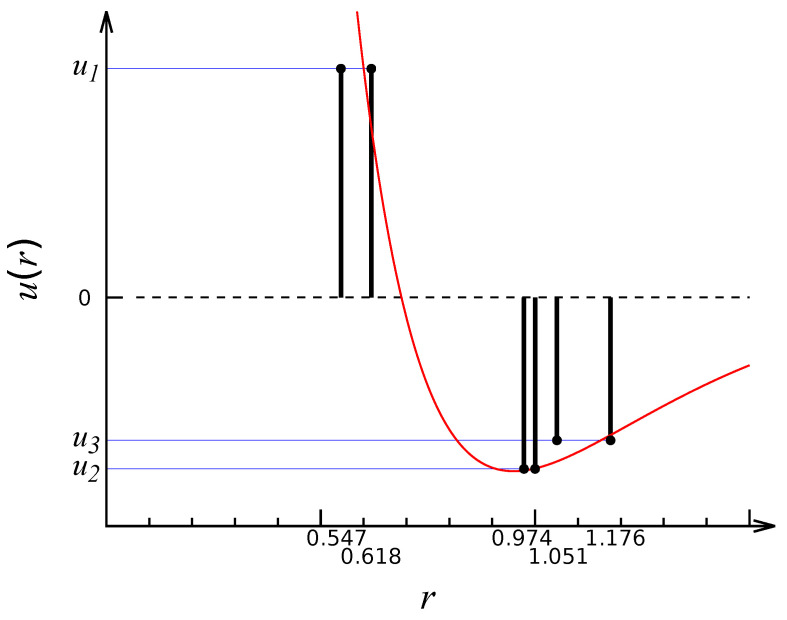
PID interaction (vertical bars at positions taken from Table 1). For the case illustrated in the picture, that is $u_{1}>0$ and $u_{2}<u_{3}<0$, the interaction is meant to represent a discrete approximant to a continuous, Morse-like potential (red line).

Calling $u_{\alpha }$ (with α=1,2,3) the strength of the interaction between α-neighbor sites, the model Hamiltonian reads

(1) $$H\left[c\right]=u_{1}\sum_{1\mathrm{NP}}c_{i}c_{j}+u_{2}\sum_{2\mathrm{NP}}c_{k}c_{l}+u_{3}\sum_{3\mathrm{NP}}c_{m}c_{n}\;,$$

where the first sum is over all distinct first-neighbor pairs (1NP) of sites, and so on. The generic profile of the interaction is sketched in Figure 2. By a suitable choice of couplings, we can represent various kinds of interparticle potential (e.g., Lennard-Jones-like or SALR—where the acronym SALR stands for “short-range attractive, long-range repulsive” [6]) and contrast them with one another in relation to the thermal behavior. In the following, the couplings are reported in ϵ units, where ϵ>0 is an arbitrary unit of energy. In turn, the temperature *T* is given in units of $\epsilon /k_{B}$, where $k_{B}$ is Boltzmann’s constant.

Once the couplings have been fixed, we compute the properties of the lattice gas in the grand canonical ensemble by running a WL simulation [7]. The WL method is designed to directly estimate (in a virtually exact manner) the density of states of the system; it is particularly suited for discrete systems with a relatively small number of degrees of freedom. In our implementation, the method performs a non-Markovian dynamics in state space by visiting *all* the available energy (E) and particle number (N) levels. It manages to achieve this by sampling a probability density proportional to the reciprocal of the density of states $g_{N,E}$. This makes the free-energy barriers invisible, meaning that all E and N values (favorable and less favorable) are visited at the same rate. In practice, $g_{N,E}$ is updated during the simulation by multiplying by a modification factor *f*, which is reduced in stages (e.g., halved) once the histogram of energies and particle numbers has become approximately flat. As time passes, the estimate of $g_{N,E}$ becomes more and more accurate, until convergence to the actual density of states eventually occurs [8,9]. It goes without saying that having a fast and effective method for reconstructing the phase diagram allows one to examine with relatively little effort the many possibilities covered by a multi-parameter model like ours.

In our WL simulations, we reduce the logarithm of *f* by a factor of two when the histogram for all values of N and E is not less than 90% of the average histogram level. The simulation is stopped when lnf becomes smaller than $10^{-7}$.

Before closing this section, we list a number of formulae relating to quantities that are needed in Section 3. Let *M* be the number of mesh sites (M=42 for the PID), *T* the temperature, μ the chemical potential, and $\beta ={\left(k_{B}T\right)}^{-1}$. From the knowledge of the density of states, the partition function immediately follows as

(2) $$\Xi =\sum_{\left\{c\right\}}e^{\beta \mu \sum_{i}c_{i}}e^{-\beta H[c]}=\sum_{N,E}g_{N,E}e^{\beta \mu N}e^{-\beta E}\;,$$

where $g_{N,E}=\sum_{\left\{c\right\}}\delta_{\sum_{i}c_{i},N}\delta_{H[c],E}$ is the total number of microstates/configurations with N particles and energy E. The grand potential $\Omega =-k_{B}T\ln\Xi$ is a function of *T* and μ, related to the pressure *P* of the system by Ω=−MP(T,μ), or $P=(k_{B}T/M)\ln\Xi$ (notice that *P* has energy dimensions). Indeed, for a system defined on a grid the volume is reduced (in units of cell volume) to the number *M* of sites. Then, the above expression of pressure derives from the assumption that the grand potential has the same volume dependence that applies for a homogeneous macroscopic system. In a finite system close to zero temperature, the slope of *P* as a function of μ undergoes a rapid increase near any phase crossover, while being roughly constant across the phase regions.

The grand canonical averages of $N=\sum_{i}c_{i}$ and E=H[c] are given by

(3) $$N=\langle N\rangle =\frac{\sum_{N,E}Ng_{N,E}e^{\beta \mu N}e^{-\beta E}}{\sum_{N,E}g_{N,E}e^{\beta \mu N}e^{-\beta E}}\;\;\;\;\;\;\mathrm{and}\;\;\;\;\;\;E\equiv \langle H\left[c\right]\rangle =\frac{\sum_{N,E}Eg_{N,E}e^{\beta \mu N}e^{-\beta E}}{\sum_{N,E}g_{N,E}e^{\beta \mu N}e^{-\beta E}}\;,$$

corresponding to the thermodynamic values of the particle number and energy, respectively. It immediately follows that

(4) $${\left.\frac{\partial P}{\partial \mu }\right|}_{T}=\frac{\langle N\rangle }{M}\equiv \rho$$

(ρ is the number density and, by definition, it is dimensionless). At fixed *T*, the density ρ is an increasing function of μ, alternating plateaus (phases) with smooth steps (phase crossovers), which are sharper at lower temperatures. While true phases cannot exist in a finite-size system, a well-defineddensity plateau indicates the resistance of the system structure to small changes in the control parameter μ at fixed *T*, which is a distinctive feature of low-temperature phases.

Two further quantities that are worth considering are the isothermal compressibility $\kappa_{T}$ and the entropy per site *s*, respectively given by

(5) $$\kappa_{T}=\frac{1}{\rho^{2}}{\left.\frac{\partial \rho }{\partial \mu }\right|}_{T}\;\;\;\;\;\;\mathrm{and}\;\;\;\;\;\;s\left(T,\mu \right)=-\frac{1}{M}{\left.\frac{\partial \Omega }{\partial T}\right|}_{\mu }={\left.\frac{\partial P}{\partial T}\right|}_{\mu }\;.$$

For systems defined on the continuum, the former expression of compressibility is equivalent to the usual definition of $\kappa_{T}$ in terms of the pressure derivative of volume, which however does not apply in the present case. An explicit expression for the entropy is

(6) $$\begin{array}{ccc} s(T,\mu ) & = & \frac{k_{B}}{M}\ln\Xi +\frac{1}{MT}\frac{\sum_{N,E}g_{N,E}\left(E-\mu N\right)e^{\beta \mu N}e^{-\beta E}}{\sum_{N,E}g_{N,E}e^{\beta \mu N}e^{-\beta E}}\\ & = & \frac{1}{MT}\left(E+MP-\mu N\right)\;, \end{array}$$

which is nothing but the Euler equation. Moreover, it easily follows from Equations (3)–(5) that

(7) $$\rho k_{B}T\kappa_{T}=\frac{\langle N^{2}\rangle -{\langle N\rangle }^{2}}{\langle N\rangle }\;.$$

Then, the stability condition $\kappa_{T}>0$ follows, either by considering the monotonicbehavior of ρ as a function of μ at fixed *T* or as a result of a positive value of $\langle N^{2}\rangle -{\langle N\rangle }^{2}=\langle {\left(N-\langle N\rangle \right)}^{2}\rangle$.

## 3. Results

We hereafter review a few cases of PID interaction, by far the most representative ones, highlighting for each the phase behavior at low temperature. We only consider integer couplings (units of ϵ), which is sufficient to exhaust the range of interesting behaviors.

**Figure 3:**
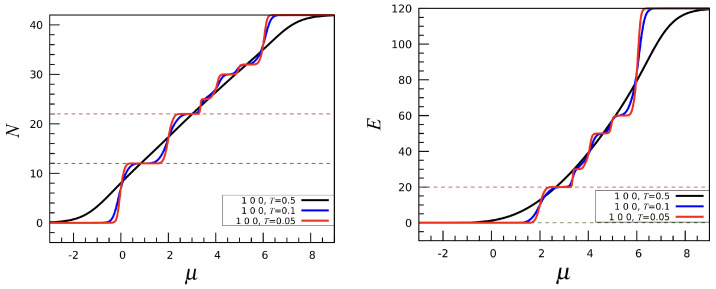
The (1, 0, 0) interaction: Particle number *N* (**left**) and energy *E* (**right**) plotted as a function of μ for three values of *T*. The dashed lines mark special values of *N* and *E* where a longer plateau occurs.

**Figure 4:**
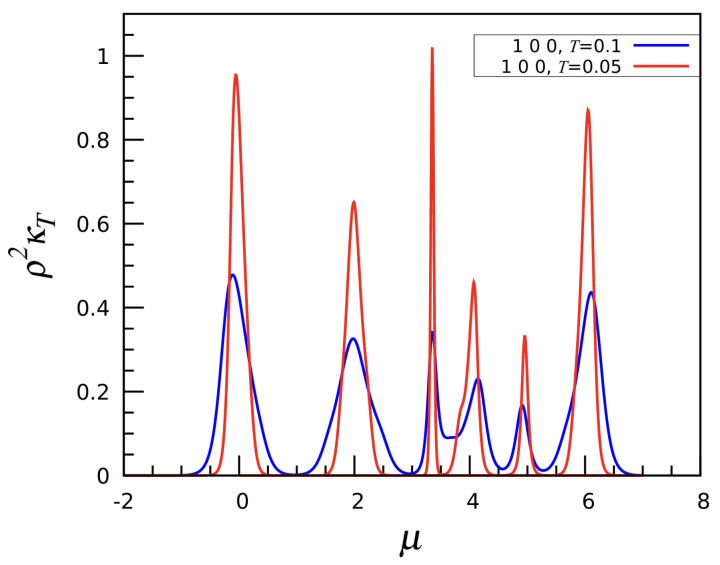
The (1, 0, 0) interaction: Isothermal compressibility $\kappa_{T}$ plotted as a function of μ for two values of *T*.

The simplest interaction is a first-neighbor repulsion: $u_{1}=1,u_{2}=u_{3}=0$ or, in short, (1, 0, 0). The profile of N(μ) is shown in the left panel of Figure 3 for three values of *T*. As further emphasized by the energy profile E(μ) plotted in the right panel, the system shows multiple “phases”, each characterized by corresponding plateaus in *N* and *E*. For negative values of μ, the most stable phase is the empty mesh. Near μ=0, a sharp “phase transition” occurs to a state with N=12 and E=0. Looking at the density of states, there are approximately $e^{4.051}\approx 57$ distinct configurations available to the system for N=12, corresponding to the many ways twelve particles can be arranged on the mesh while avoiding being mutual nearest neighbors. In most of these configurations, the particles form an irregular icosahedron; in the specific case where all twelve fivefold sites are occupied, a regular icosahedron is formed instead, while the empty sites draw an ID. Further increasing the chemical potential results in a phase transition to the N=22 phase, located at μ≈2. For this value of *N*, the configurations with minimum energy are only “lanes” and “worms”, i.e., one-dimensional lamellae, as better explained below. At still larger μ, the system crosses three other phases with 25,30, and 32 occupied sites. However, no clearly recognizable pattern is found in these cases. Finally, above μ≈6 all the 42 sites of the mesh are occupied. Each phase crossover is marked by a narrow $\kappa_{T}$ maximum; see Figure 4.

**Figure 5:**
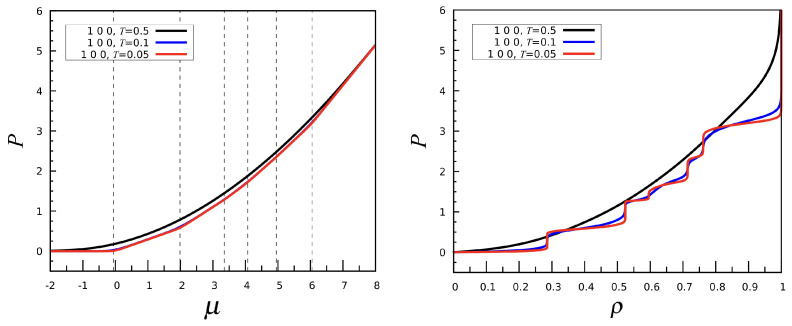
The (1, 0, 0) interaction: *P* vs. μ (**left**) and *P* vs. ρ (**right**) for three values of *T*.

**Figure 6:**
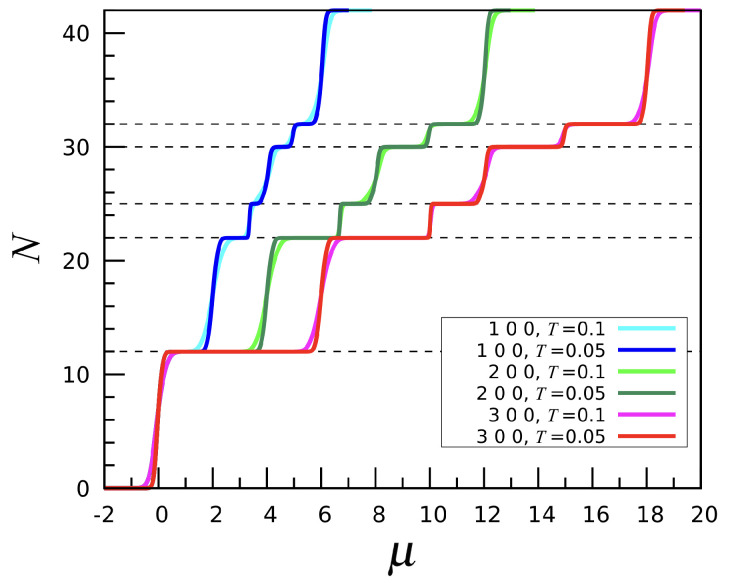
The (n,0,0) interaction, with n=1,2,3: Particle number *N* plotted as a function of μ for two values of *T*.

The pressure P(μ) can be computed either by integrating the number density ρ(μ) (see Equation (4)) or directly from the density of states, with negligible differences between the two routes. Since the density is an increasing step-like function of μ at low *T*, we expect the slope of the pressure curve to undergo a sharp increase at each transition. This is confirmed in Figure 5 (left), where the dotted lines mark the location of the $\kappa_{T}$ peaks. For the same three temperatures, the equation of state P(ρ) is plotted in the right panel of Figure 5. In this picture, we see the characteristic plateaus of *P* expected for a macroscopic system undergoing a sequence of first-order phase transitions. Finally, we show in Figure 6 that increasing the strength of the first-neighbor repulsion just shifts the phase transitions to higher values of μ.

**Figure 7:**
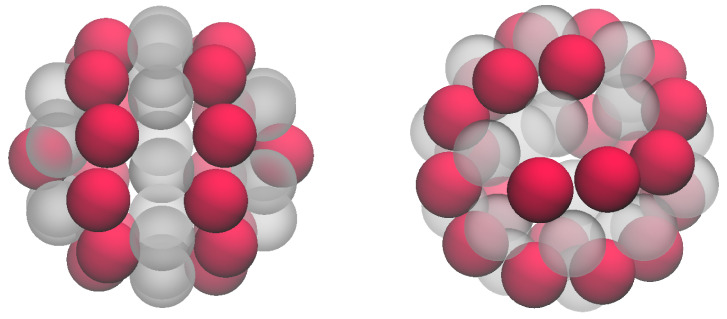
The (1, 0, 0) interaction: Two typical configurations (with red spheres denoting the occupied sites and grey spheres the empty sites) for N=22 and E=20; this value of *E* is the minimum possible for N=22.

As mentioned above, the system displays an unusual pattern for N=22. In Figure 7, we show two typical states with minimum energy. On the left, particles form two parallel rings (lanes) with 10 particles each, while two more particles are located at opposite poles. On the right, two particle worms (sort of truncated spirals) are wrapped around each other. It is noteworthy that such complex states can emerge by just adopting nearest-neighbor repulsion in the PID model. Thin stripes in the ANNNI model [10] are somewhat analogous to lanes in a lattice-gas model but, at variance with the present case, they require an anisotropic interaction to occur. A simple isotropic potential in two dimensions where lanes are observed is the core–corona potential studied in Ref. [11]. Lanes and worms have been seen in other fluids of particles confined in a spherical surface, but only by using more elaborate interactions [12,13,14]. Lanes have also been described for a triangular lattice gas with an interaction featuring a soft barrier and a longer-range attraction [15]. It is interesting that the PID model can reproduce patterns that are present in a number of geometrically constrained systems.

When we include a second-neighbor attraction, while still keeping the first-neighbor repulsion, the worm-like patterns are suppressed, giving way to more irregular structures. These cases also include interactions featuring a second-neighbor attraction and a weaker third-neighbor attraction (Lennard-Jones-like interactions).

**Figure 8:**
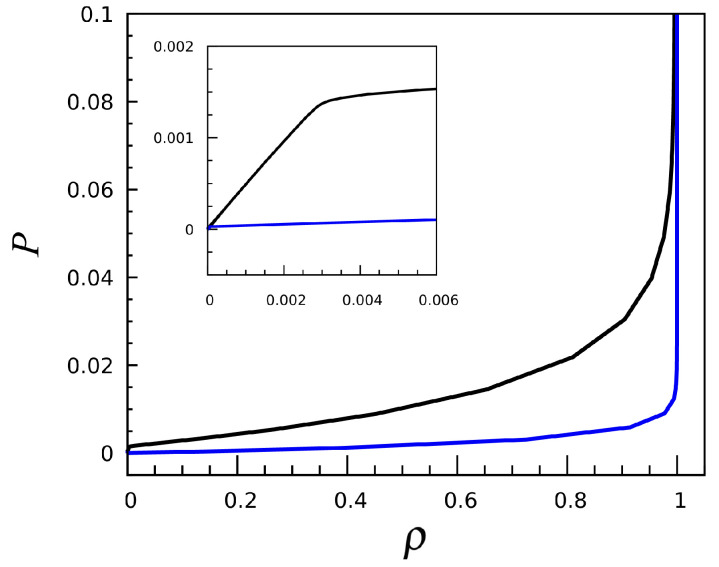
The (−1,0,0) interaction: Equation of state for T=0.3 (blue line) and 0.5 (black line). In the inset, the low-density region is magnified for clarity.

Since the constraint of single-site occupancy plays the same role as a hard core, we can also represent a Lennard-Jones-like potential by taking negative values for $u_{1}$ (the potential well) and less negative (or null) values for $u_{2}$ and $u_{3}$. For instance, when $u_{1}=-1$ and $u_{2}=u_{3}=0$, the interaction favors particle aggregation; beyond a specific μ value, the initially empty mesh becomes completely filled, without passing through intermediate stages. This phenomenology corresponds to the solid–vapor transition near zero temperature. This is demonstrated in Figure 8, where we plot the equation of state for T=0.3 and 0.5. In a wide interval of densities, the pressure changes only slightly, a behavior recalling two-phase separation. Exactly the same outcome is found for $u_{1}=-1,u_{2}=-1$, and $u_{0}=0$, which mimics a square-well potential.

**Figure 9:**
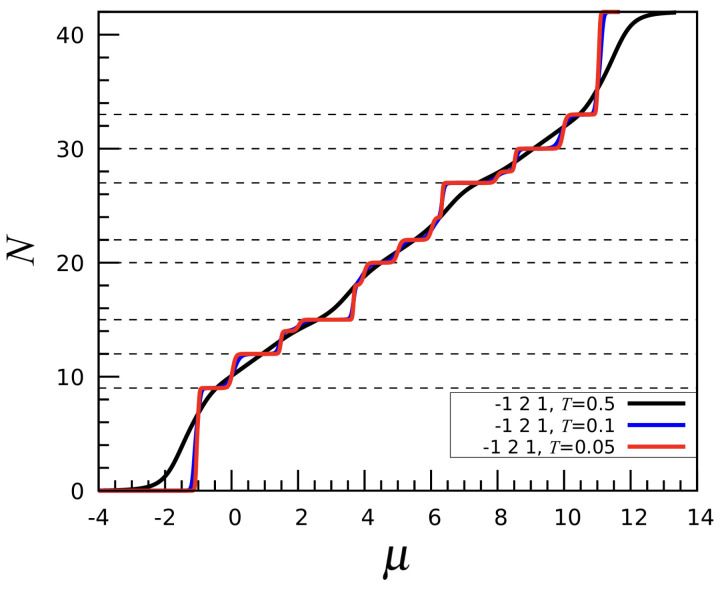
The (−1,2,1) interaction: *N* vs. μ for three temperatures (in the legend).

**Figure 10:**
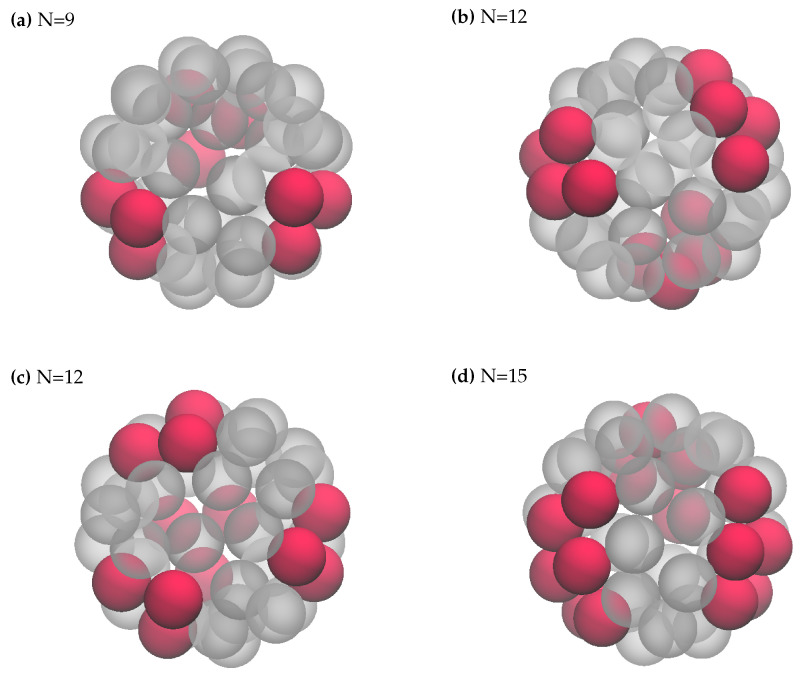
The (−1,2,1) interaction: Typical minimum-energy states for various *N* values. These configurations are reminiscent of cluster crystals.

By combining a first-neighbor attraction with a longer-range (second- and third-neighbor) decreasing repulsion we obtain the discrete analog of an SALR potential [6]. For instance, setting $u_{1}=-1,u_{2}=2,u_{3}=1$, the particle number profile is as depicted in Figure 9. Due to the short-range attraction, this interaction promotes particle aggregation at low temperatures, while at the same time discouraging the formation of a solid (that is, of a completely filled mesh). However, at sufficiently high μ a dense phase inevitably forms. Upon increasing the chemical potential (or the density), the system would thus cross a region of thermodynamic space characterized by a sequence of cluster-crystal microphases. Figure 10 shows four distinct cluster-crystal configurations, illustrating how the clusters grow in size when increasing the density. The self-assembly of simple molecules into clusters that are regularly distributed on a spherical surface can be a means for the synthesis of patchy particles [16]. Eventually, for N=20, the clusters merge together in a large connected droplet. For N>21, the roles of “vapor” and clusters are interchanged, resulting in configurations which are the reverse images of the ones described earlier. This behavior is consistent with the profile of N(μ), which is indeed symmetric around N=21. We provide in Appendix A a theoretical argument for the symmetry observed in N(μ) for the (−1,2,1) model, which is absent in the cases previously considered.

**Figure 11:**
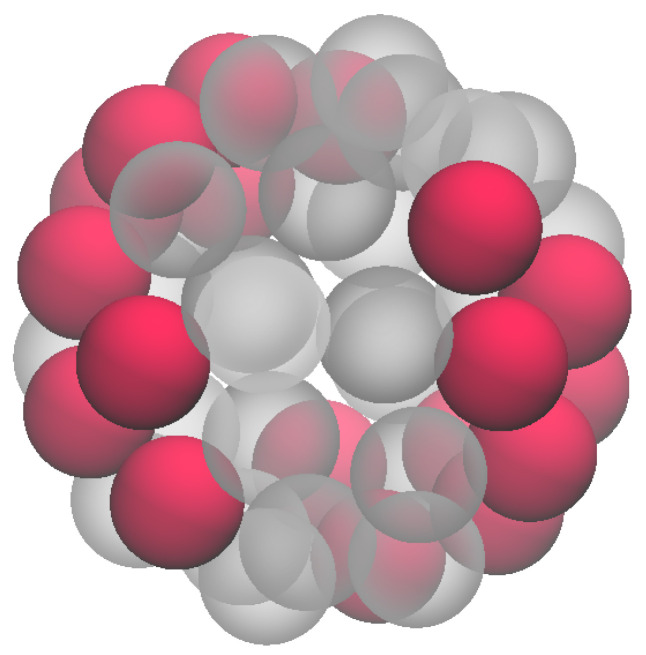
The (−1,1,0) interaction: Minimum-energy configuration for N=18.

**Figure 12:**
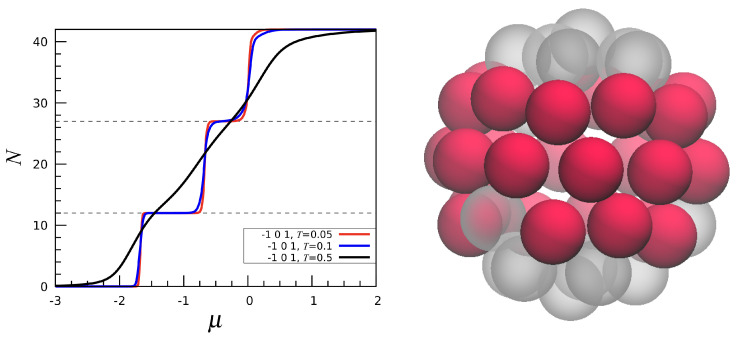
The (−1,0,1) interaction. (**Left**): *N* vs. μ for three values of *T*. (**Right**): Minimum-energy configuration for N=27.

Cluster-crystal patterns have also been found in triangular lattice gases in the presence of an SALR interaction [17,18], as well as in various systems of softly repulsive particles confined on a spherical surface [19,20,21,22]. It is worth stressing that lowest-energy configurations with clusters are also seen in the simpler (−1,1,0) model, though the clusters now contain no less than four particles. In addition, we observe a “tennis ball” (see Figure 11). Also interesting is the (−1,0,1) model, another SALR interaction, where two plateaus are present in N(μ) (see Figure 12 left). For N=12, the most stable configuration shows two clusters with six particles each, lying at diametrically opposed poles. Instead, for N=27 the occupied sites form a ribbon around the sphere (Figure 12 right).

**Figure 13:**
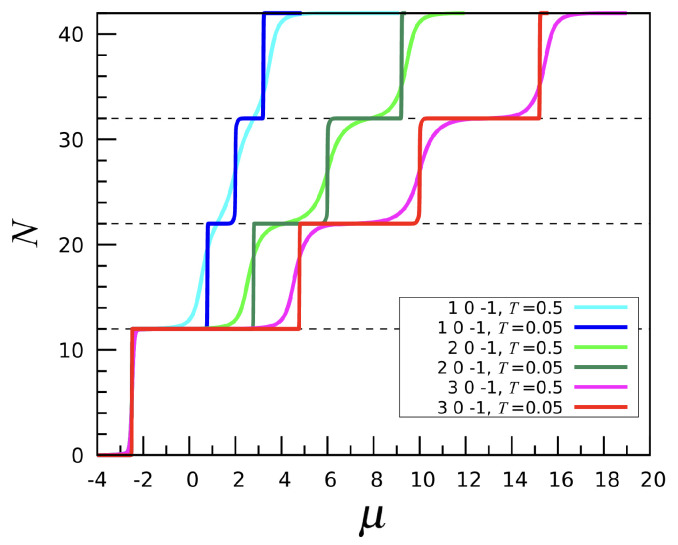
The (n,0,−1) interaction, with n=1,2,3: Particle number *N* plotted as a function of μ for two temperatures (in the legend).

**Figure 14:**
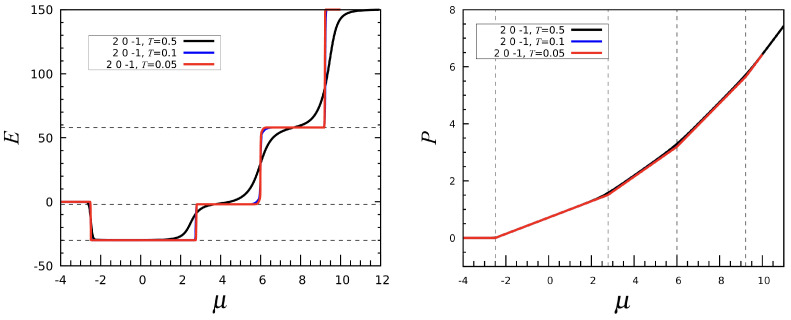
The (2,0,−1) interaction. (**Left**): Energy *E* plotted as a function of μ for three temperatures. The dashed lines mark the energies of the three phases with N=12,22, and 32, respectively. (**Right**): *P* vs. μ for the same temperatures. Here, the dashed lines are drawn at the location of the transitions.

Introducing a third-neighbor attraction on top of a first-neighbor repulsion and assuming a weaker or null second-neighbor attraction does not drastically alter the system behavior compared to (1, 0, 0), but some notable differences arise. In Figure 13, we plot the number *N* of particles as a function of μ for varying first-neighbor repulsion and a fixed third-neighbor attraction strength $u_{3}=-1$, while $u_{2}$ is set to zero. As seen in the picture, the system undergoes a sequence of sharp phase transitions as μ increases. For largenegative μ values, the stable phase is the empty mesh. Upon increasing μ, the N=12 phase eventually emerges, with particles located at fivefold sites. As shown in the left panel of Figure 14 (referring to the (2,0,−1) interaction), the icosahedral phase is accompanied by a deep energy minimum; this happens because two particles located in the nearest fivefold sites are third neighbors of each other. The next N=22 phase is again characterized by a double-spiral pattern, while lanes are absent. Admittedly, the addition of a third-neighbor attraction causes the lanes and double spirals to separate in energy, with the latter being preferred because of a lower energy, i.e., a larger number of third-neighbor particle pairs. As μ grows further, we meet the N=32 phase, but, as for (1, 0, 0), no regular pattern is observed. Eventually, for still larger μ the mesh becomes completely filled.

As usual, every phase crossover is marked by a peak in the isothermal compressibility $\kappa_{T}$. In Figure 14 (right), we show the pressure as a function of μ. The changes occurring in the slope of P(μ) at the phase transitions are quite abrupt at T=0.05.

**Figure 15:**
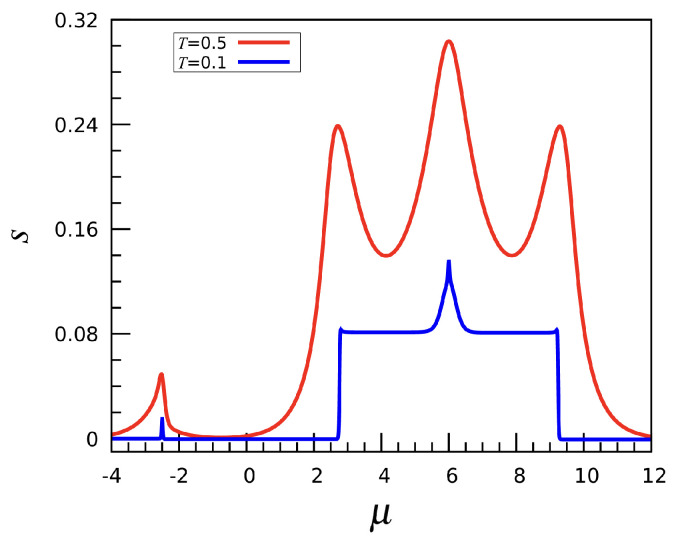
The (2,0,−1) interaction: Entropy per site *s* plotted as a function of μ at T=0.1 and 0.5.

Finally, it is worth commenting on the μ-dependence of the entropy for the (2,0,−1) case; see Figure 15. To compute *s* we can either use Equation (5) or perform the numerical derivative of *P* with respect to *T*; see Equation (4). We have verified that both results are identical. The entropy shows peaks in coincidence with all phase transitions, while assuming a larger mean value within the N=22 and N=32 phases. Eventually, *s* drops to zero when the mesh becomes completely filled. In order to rationalize this behavior, it suffices to consider that *s* is a measure of the multiplicity of a macrostate [23]. Larger *s* values occur for those (T,μ) values for which the number of microstates with high statistical weight is larger as well.

We have investigated other sets of couplings with a minimum at third-neighbor distances, such as (1,−0.5,−1),(1.5,−0.5,−1), and (2,−0.5,−1), but no major changes relative to the previous case are observed.

**Figure 16:**
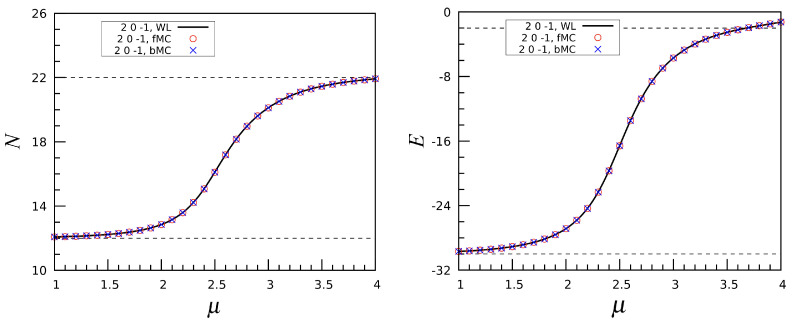
The (2,0,−1) interaction: Number of particles *N* (**left**) and energy *E* (**right**), plotted as a function of μ at T=0.5. A comparison is made between a forward MC (fMC) simulation, a backward MC (bMC) simulation, and the WL simulation. Notice how the red and blue symbols are perfectly overlapped.

**Figure 17:**
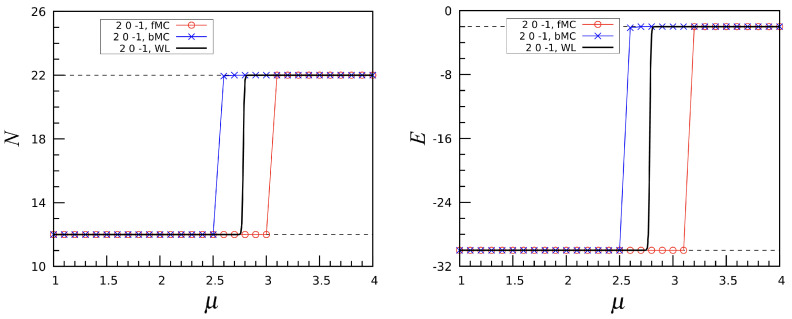
The (2,0,−1) interaction: Same as Figure 16, but for T=0.05.

To gain a deeper understanding of the nature of the phase crossovers in the PID model, we have performed an additional analysis in terms of grand canonical Monte Carlo (MC) simulations, using the standard Metropolis algorithm. We focus on the transition from N=12 to N=22 in the (2,0,−1) model. In Figure 16, we display the number of particles and energy as functions of μ for T=0.5. Both quantities were determined through a sequence of MC runs, starting from μ=1 (in the icosahedral phase) and incrementing the chemical potential in steps of 0.1 up to μ=4 (well within the double-spiral phase). At every μ, starting from the last configuration generated at the previous μ value in the sequence, we performed $10^{6}$ cycles (MC moves per site) to equilibrate the sample, followed by $5\times 10^{6}$ cycles in the production run. Similarly, a backward MC simulation was carried out, starting from a double-spiral state at μ=4 and reducing μ in steps, until we came back to the icosahedral phase. The results of these two simulations align perfectly with one another and with the data obtained by the Wang–Landau algorithm. However, things change drastically when the temperature is very low, for example, T=0.05. As shown in Figure 17, the *N* and *E* curves obtained from the forward and backward MC simulations now differ significantly, showing clear evidence of hysteresis. This outcome indicates that at very low temperature the phase crossovers identified in the PID model have the same characteristics of first-order phase transitions. In conclusion, when *T* is very low the Metropolis MC method experiences serious problems in sampling the equilibrium distribution, being unable to precisely locate the phase transition. In contrast, the WL algorithm offers an accurate estimate of the transition point.

Summing up, a number of regular patterns (icosahedral crystals and “lanes”) can be induced on a spatial grid formed by the vertices of a PID, in spite of the geometric frustration induced by the curvature of the mesh. On a curved mesh, extended triangular order is impossible; yet, for suitable interactions even a finite lattice-gas system finds a way to minimize energy in a symmetric fashion. Moreover, various types of complex “fluid” structures, such as worm-like and cluster aggregates, can be promoted at low temperature in the presence of an SALR interaction reaching the third-neighbor distance.

## 4. Conclusions

We have studied a lattice-gas model on a semiregular mesh obtained by projecting the vertices of a convex polyhedron, the pentakis icosidodecahedron (PID), onto a spherical surface. In this model, interactions range from first to third neighbors. Due to its relatively small size (only 42 sites in the mesh), the model is suitable for an exact investigation of thermodynamic behavior by the Wang–Landau method. Since it is finite, the system cannot exhibit sharp phases and phase transitions, though at low temperature we observe neat crossovers in the statistical properties, having characteristics similar to phase transitions in a macroscopic system.

Despite its simplicity, the collective behavior of the PID lattice gas is quite rich: depending on the energy couplings, the model shows (in rudimentary terms) the same phenomenology of popular interactions in three dimensions, such as the soft-core repulsion, the Lennard-Jones potential, and the SALR potential. In particular, we identify “crystals” (of icosahedral symmetry), “cluster crystals”, and worm-like structures (“lanes” and “spirals”). Unveiling the microscopic mechanism behind the formation of regular patterns on a polyhedral surface may favor the interpretation of experiments on colloidal aggregation over curved surfaces, such as oil droplets [24]. In the near future we plan to extend the study to triangle meshes of increasing complexity, with the purpose of investigating the cause-and-effect relationship between interaction law and emergent structure in other solvable models.

## Figures and Tables

**Table 1:** Chord distances from a vertex to its neighbors, distinguishing a sixfold vertex from a fivefold one (the radius of the circumscribed sphere is 1). The number within parentheses indicates how many neighbors of each type are present.

Neighbor	Sixfold Vertex	Fivefold Vertex
First	0.546533 (2), 0.618034 (4)	0.546533 (5)
Second	0.973929 (2), 1 (4)	0.973929 (5)
Third	1.175571 (4)	1.051462 (5)

## Data Availability

Dataset available on request from the authors.

## Appendix A. Proof of a Symmetry Property

The curve N(μ) in Figure 9 is manifestly symmetric around N=M/2=21, whereas no analogous inversion symmetry appears in other cases (see Figure 6 and Figure 13). We hereafter provide an explanation for this fact.

The mentioned symmetry corresponds to the existence of a chemical potential value $\mu_{0}$ such that

(A1) $$N\left(\mu_{0}+\Delta \mu \right)=M-N\left(\mu_{0}-\Delta \mu \right)\;.$$

By working out the right-hand side of Equation (A1), we successively obtain

(A2) $$\begin{array}{ccc} M-N(\mu ) & = & \langle M-N\rangle \left(\mu \right)=\frac{\sum_{\left\{c\right\}}\left(M-\sum_{i}c_{i}\right)e^{\beta \mu \sum_{i}c_{i}-\beta H\left[c\right]}}{\sum_{\left\{c\right\}}e^{\beta \mu \sum_{i}c_{i}-\beta H\left[c\right]}}\\ & = & \frac{\sum_{\left\{c\right\}}\left(\sum_{i}\left(1-c_{i}\right)\right)e^{\beta \mu \sum_{i}c_{i}-\beta H\left[c\right]}}{\sum_{\left\{c\right\}}e^{\beta \mu \sum_{i}c_{i}-\beta H\left[c\right]}}\;. \end{array}$$

Using the substitution $c_{i}\to 1-c_{i}$ for all *i*, we arrive at

(A3) $$M-N\left(\mu \right)=\frac{\sum_{\left\{c\right\}}\left(\sum_{i}c_{i}\right)e^{\beta \mu \sum_{i}\left(1-c_{i}\right)-\beta H\left[1-c\right]}}{\sum_{\left\{c\right\}}e^{\beta \mu \sum_{i}\left(1-c_{i}\right)-\beta H\left[1-c\right]}}\;.$$

Now, we need to compute H[1−c]:

(A4) $$\begin{array}{c} H\left[1-c\right]=u_{1}\sum_{1\mathrm{NP}}\left(1-c_{i}\right)\left(1-c_{j}\right)+\ldots =u_{1}\left(\sum_{1\mathrm{NP}}1-2\sum_{1\mathrm{NP}}c_{i}+\sum_{1\mathrm{NP}}c_{i}c_{j}\right)+\ldots \;, \end{array}$$

omitting similar terms containing $u_{2}$ and $u_{3}$. We have

(A5) $$\begin{array}{c} \sum_{1\mathrm{NP}}1=\sum_{2\mathrm{NP}}1=\frac{1}{2}\left(12\cdot 5+30\cdot 6\right)=120\;;\;\;\;\;\;\;\sum_{3\mathrm{NP}}1=\frac{1}{2}\left(12\cdot 5+30\cdot 4\right)=90\;. \end{array}$$

More tricky is the evaluation of $\sum_{1\mathrm{NP}}c_{i}$. Calling NN_*i*_ the nearest neighbors of site *i*, 5s the fivefold sites, and 6s the sixfold sites, we obtain

(A6) $$\sum_{1\mathrm{NP}}c_{i}=\frac{1}{2}\sum_{i}c_{i}\sum_{j\in {\mathrm{NN}}_{i}}1=\frac{1}{2}\left(\sum_{5s}c_{i}\cdot 5+\sum_{6s}c_{i}\cdot 6\right)=\frac{5}{2}\sum_{i}c_{i}+\frac{1}{2}\sum_{6s}c_{i}\;.$$

Similarly,

(A7) $$\sum_{2\mathrm{NP}}c_{i}=\frac{5}{2}\sum_{i}c_{i}+\frac{1}{2}\sum_{6s}c_{i}\;\;\;\;\;\;\mathrm{and}\;\;\;\;\;\;\sum_{3\mathrm{NP}}c_{i}=\frac{5}{2}\sum_{i}c_{i}-\frac{1}{2}\sum_{6s}c_{i}$$

The last terms in Equations (A6) and (A7) are configuration-dependent in a complicated way. However, if $u_{1}+u_{2}=u_{3}$, the contribution from those terms vanish and we obtain

(A8) $$H\left[1-c\right]=30\left(4u_{1}+4u_{2}+3u_{3}\right)-5\left(u_{1}+u_{2}+u_{3}\right)\sum_{i}c_{i}+H\left[c\right]\;.$$

Under the condition $u_{1}+u_{2}=u_{3}$, Equation (A3) then becomes

(A9) $$\begin{array}{ccc} M-N(\mu ) & = & \frac{\sum_{\left\{c\right\}}\left(\sum_{i}c_{i}\right)e^{-\beta \mu \sum_{i}c_{i}}e^{5\beta \left(u_{1}+u_{2}+u_{3}\right)\sum_{i}c_{i}}e^{-\beta H[c]}}{\sum_{\left\{c\right\}}e^{-\beta \mu \sum_{i}c_{i}}e^{5\beta \left(u_{1}+u_{2}+u_{3}\right)\sum_{i}c_{i}}e^{-\beta H[c]}}\\ & = & \frac{\sum_{\left\{c\right\}}\left(\sum_{i}c_{i}\right)e^{\beta \left(5\left(u_{1}+u_{2}+u_{3}\right)-\mu \right)\sum_{i}c_{i}}e^{-\beta H[c]}}{\sum_{\left\{c\right\}}e^{\beta \left(5\left(u_{1}+u_{2}+u_{3}\right)-\mu \right)\sum_{i}c_{i}}e^{-\beta H[c]}}\\ & = & N\left(5(u_{1}+u_{2}+u_{3})-\mu \right)\;. \end{array}$$

Upon comparing Equations (A1) and (A10), we can make the following identification:

(A10) $$\mu =\mu_{0}-\Delta \mu \;\;\;\;\;\;\mathrm{and}\;\;\;\;\;\;5\left(u_{1}+u_{2}+u_{3}\right)-\mu =\mu_{0}+\Delta \mu \;,$$

which is tantamount to

(A11) $$\mu_{0}=\frac{5}{2}\left(u_{1}+u_{2}+u_{3}\right)\;\;\;\;\;\;\mathrm{and}\;\;\;\;\;\;\Delta \mu =\frac{5}{2}\left(u_{1}+u_{2}+u_{3}\right)-\mu \;.$$

In particular, for the model (−1,2,1) the density curve must be symmetric around the point with μ=5 and N=21, which is indeed the case.
